# The complete chloroplast genome sequence of *Coix lacryma-jobi* L. (Poaceae), a cereal and medicinal crop

**DOI:** 10.1080/23802359.2018.1507653

**Published:** 2018-10-23

**Authors:** Sang-Ho Kang, Hyun Oh Lee, Myung Ju Shin, Nam-Hoon Kim, Beom-Soon Choi, Manu Kumar, Asjad Ali, Sang-Choon Lee, Chang-Kug Kim

**Affiliations:** a Genomics Division, National Institute of Agricultural Sciences, Rural Development Administration, Jeonju, Republic of Korea;; b Phyzen Co, Seongnam, Republic of Korea;; c Department of Bioindustry and Bioresource Engineering, Plant Engineering Research Institute, Sejong University, Seoul, Republic of Korea;; d Southern Cross Plant Science, Southern Cross University, Lismore, Australia

**Keywords:** *Coix lacryma-jobi*, poaceae, chloroplast genome, cereal crop, medicinal plant

## Abstract

*Coix lacryma-jobi* is a cereal and medicinal crop belonging to the Poaceae family. This study characterized complete chloroplast genome sequence of a Korean cultivar Johyun of *C. lacryma-jobi* var. *ma-yuen* through the *de novo* hybrid assembly with Illumina and PacBio genomic reads. The chloroplast genome is 140,863 bp long and composed of large single copy (82,827 bp), small single copy (12,522 bp), and a pair of inverted repeats (each 22,757 bp). A total of 123 genes including 87 protein-coding genes, 32 tRNA genes, and four rRNA genes were predicted in the genome. Phylogenetic analysis confirmed a close relationship of *C. lacryma-jobi* with species in the Panicoideae subfamily of the Poaceae family.

*Coix lacryma-jobi* is an annual herb, called also as adlay or Job’s tears, belonging to the Panicoideae subfamily of the Poaceae family and cultivated in tropical and subtropical regions of the world. *C. lacryma-jobi* includes many variants such as *C. lacryma-jobi* var. *ma-yuen* with soft shell (Chen et al. 2006). In Korea, *C. lacryma-jobi* var. *ma-yuen* is called as Yulmu and has been cultivated as a cereal and medicinal crop (Park and Lee 1999; Kim et al. 2000; Lee et al. 2002; RDA 2013). Several genomic resources have been reported for *C. lacryma-jobi*, however, those are insufficient for breeding and molecular study of this species, compared to other Panicoideae species such as maize and sorghum whose genomes have been sequenced (Jiao et al. 2017; McCormick et al. 2018). On this account, this study characterized the complete chloroplast genome of *C. lacryma-jobi* var. *ma-yuen*.

A Korean cultivar Johyun of *C. lacryma-jobi* var. *ma-yuen* was bred by Gyeonggido Agricultural Research and Extension Services (http://nongup.gg.go.kr/, cultivar no. SJ9203) in Korea (Jang et al. 2005) and registered in Korea Seed and Variety Service (https://www.seed.go.kr) under registration no. 05-0020-4. Genomic DNA was extracted from leaves of the cultivar and sequenced using the Illumina HiSeq2500 and PacBio RSII platforms. High quality reads including Illumina paired-end reads of about 5.9 Gb and PacBio reads of about 9.9 Gb were *de novo* hybrid assembled using SPAdes (ver. 3.9.0, http://cab.spbu.ru/software/spades/) and then chloroplast contigs were selected and sorted by comparison with reported *C. lacryma-jobi* chloroplast genome sequence (NC_013273, Leseberg and Duvall 2009). The selected contigs were merged and gap-filled by a series of read mapping and then chloroplast genome was completed as described by Kim et al. (2015).

The complete chloroplast genome (GenBank Accession no. MH558672) is 140,863 bp long and consists of large single copy (LSC, 82,827 bp), small single copy (SSC, 12,522 bp), and a pair of inverted repeats (IRa and IRb, each 22,757 bp). A total of 123 genes were predicted in the genome, including 87 protein-coding genes, 32 tRNA genes, and four rRNA genes.

Phylogenetic analysis of *C. lacryma-jobi* var. *ma-yuen* cv. Johyun with other taxa was performed using a Maximum Likelihood (ML) method with whole chloroplast genome sequences and revealed that *C. lacryma-jobi* var. *ma-yuen* cv. Johyun formed a group with species in the Andropogoneae tribe of the Panicoideae subfamily and was much closed (∼99% identity) to the studied *C. lacryma-jobi* species (Figure 1). This phylogenetic relationship was consistent with previous studies (Leseberg and Duvall 2009; Teerawatananon et al. 2011).

**Figure 1.**
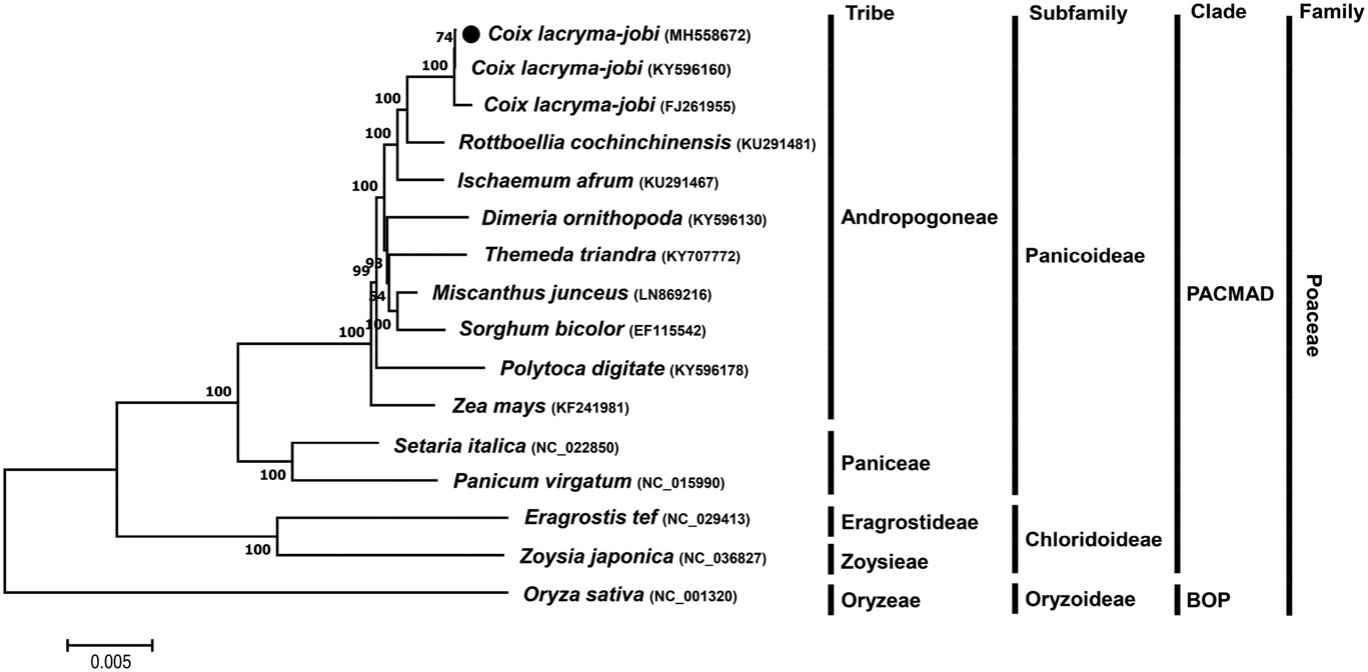
ML phylogenetic tree of chloroplast genomes of *C. lacryma-jobi* var.*ma-yuen* cv. Johyun and other Poaceae species. Whole chloroplast genome sequences were multiple-aligned using MAFFT (http://mafft.cbrc.jp/alignment/server/index.html) and used to generate phylogenetic tree by MEGA 7.0 (Kumar et al., 2016). The bootstrap support values (>50%) from 1000 replicates are indicated on the nodes. GenBank accession nos. of chloroplast genome sequences used for this tree are indicated within parentheses.
